# Elevated ATGL in colon cancer cells and cancer stem cells promotes metabolic and tumorigenic reprogramming reinforced by obesity

**DOI:** 10.1038/s41389-021-00373-4

**Published:** 2021-11-29

**Authors:** Rida Iftikhar, Harrison M. Penrose, Angelle N. King, Joshua S. Samudre, Morgan E. Collins, Alifiani B. Hartono, Sean B. Lee, Frank Lau, Melody Baddoo, Erik F. Flemington, Susan E. Crawford, Suzana D. Savkovic

**Affiliations:** 1 Department of Pathology and Laboratory Medicine, Tulane University School of Medicine, New Orleans, LA USA; 2 Department of Surgery, Louisiana State University Health Sciences Center, New Orleans, LA USA; 3 NorthShore University Research Institute, Affiliate of University of Chicago Pritzker School of Medicine, Evanston, IL USA

**Keywords:** Cancer, Stem cells

## Abstract

Obesity is a worldwide epidemic associated with increased risk and progression of colon cancer. Here, we aimed to determine the role of adipose triglyceride lipase (ATGL), responsible for intracellular lipid droplet (LD) utilization, in obesity-driven colonic tumorigenesis. In local colon cancer patients, significantly increased ATGL levels in tumor tissue, compared to controls, were augmented in obese individuals. Elevated ATGL levels in human colon cancer cells (CCC) relative to non-transformed were augmented by an obesity mediator, oleic acid (OA). In CCC and colonospheres, enriched in colon cancer stem cells (CCSC), inhibition of ATGL prevented LDs utilization and inhibited OA-stimulated growth through retinoblastoma-mediated cell cycle arrest. Further, transcriptomic analysis of CCC, with inhibited ATGL, revealed targeted pathways driving tumorigenesis, and high-fat-diet obesity facilitated tumorigenic pathways. Inhibition of ATGL in colonospheres revealed targeted pathways in human colonic tumor crypt base cells (enriched in CCSC) derived from colon cancer patients. In CCC and colonospheres, we validated selected transcripts targeted by ATGL inhibition, some with emerging roles in colonic tumorigeneses (ATG2B, PCK2, PGAM1, SPTLC2, IGFBP1, and ABCC3) and others with established roles (MYC and MUC2). These findings demonstrate obesity-promoted, ATGL-mediated colonic tumorigenesis and establish the therapeutic significance of ATGL in obesity-reinforced colon cancer progression.

## Introduction

Obesity affects half a billion individuals in both the industrialized and developing world [1, 2]. This obesity epidemic increases the risk and progression of cancer in different tissue, including the colon [3, 4]. Obese colon cancer patients have prevalent tumor recurrence and resistance to chemotherapy, both in part due to cancer stem cell populations [4, 5]. Therefore, there is an excessive, unmet demand in understanding how obesity facilitates colonic tumorigenesis and recurrence in order to develop more effective treatment options.

Colon cancer, the second leading cause of cancer-related death worldwide [6], is initiated and driven, in part, by metabolic remodeling [7–10]. With tumorigenesis, lipid remodeling drives cancer cell signaling and provides building blocks for new cancer cells, along with fuel for their growth [8, 11–13]. These well-orchestrated processes may rely on the dynamics of intracellular organelles known as lipid droplets (LDs) [14–16]. LDs harbor intracellular lipids and were initially studied in fat-storing tissue [17, 18]. They are comprised of a neutral lipid core, composed mainly of triglycerides, and surrounded by perilipin protein [19, 20]. LD’s dynamics, including lipogenesis and lipolysis, play critical roles in cellular homeostasis [19, 20]. We, and others, have demonstrated that LDs are substantially elevated in human and mouse colonic tumors and facilitate the growth of colon cancer cells [21–24]. Human colon cancer cells have different capacities for LDs accumulation, which may influence their response to therapies [22, 24, 25]. Further, we have identified LD’s negative regulatory loop with transcription factor FOXO3 that involves multiple pathways [22, 24, 26]. Emerging findings show the critical roles of LDs and FOXO3 in metabolic reprogramming of cancer stem cells behavior [27–29], the mechanisms of which are not yet well understood.

Adipose triglyceride lipase (ATGL), otherwise known as PNPLA2, is an LD-associated rate-limiting enzyme exclusively responsible for LDs utilization by breaking down their stored triglycerides [30, 31]. ATGL is primarily expressed in adipose tissue but is also found in other tissue, including the colon [32]. In adipocytes, ATGL activity is additionally regulated by the activator Abhd5 (CGI-58) and inhibitor G0S2 [33, 34]. In non-fat-storing cells, Abhd5 and G0S2 can function independently from ATGL and drive tumorigenesis [35–39]. Moreover, elevated ATGL is associated with enhanced breast and pancreatic tumor growth and invasion [40, 41], whereas ATGL deficiency in mice can lead to spontaneous lung cancer [42, 43]. A recent study showed significantly elevated ATGL levels in colonic tumor tissue relative to matched control in patients from China [44]. We hypothesize that obesity increased ATGL-mediated LDs utilization, thus facilitating colon cancer progression through metabolic and tumorigenic changes in colon cancer cells and colon cancer stem cells.

Here, we found in local colon cancer patients that increased levels of ATGL in tumor tissue, compared to matched control, were augmented by obesity. Blockade of ATGL in colon cancer cells and colonospheres, enriched in colon cancer stem cells, inhibited growth. Further, blockade of ATGL targeted metabolic and growth pathways in colon cancer cells and colonospheres. These findings identify a novel mechanism of increased ATGL-mediated LDs utilization in colon cancer progression and establish a platform for new diagnostic and therapeutic importance in colonic tumorigenesis, especially when driven by obesity.

## Materials and methods

### Human colon cancer samples

Primary human colon tumor tissue samples were obtained from local patients through the Louisiana Cancer Research Consortium Biospecimen core (www.louisianacancercenter.org), processed in compliance with NIH regulations and institutional guidelines. Human colonic tumor tissue was acquired from ten patients, three female and seven male, ages 39–67 of both African American (*n* = 5) and Caucasian (*n* = 5) ethnic backgrounds. Body mass index (BMI), as calculated (kilogram/meters^2^), ranged from 23 to 40 and was determined on de-identified patient tumor samples at the time of tissue collection. The de-identified patient samples, used in this study, were approved by the institutional review board (IRB) at Tulane University, which waived the requirement for informed consent for sample collection.

Publicly available transcriptomes obtained from colon cancer patients included control (*n* = 43) and colonic tumors samples (*n* = 72) (GSE20916 and GSE62322). Further, publicly available transcriptomes from human colonic tumor crypts relative to control crypts from colon cancer patients (*n* = 5) (GSE20916). Additionally, we utilized publicly available transcriptomic data obtained from colon cancer patients (TCGA), normal colon (*n* = 41), and tumors (*n* = 457). These data are acquired using NCBI’s GEO2R.

### High-fat-diet obese mice and colonic tumors model

Transcriptomic data from colon and colonic tumors of high-fat-diet obese mice (*n* = 3 for each group) were acquired before [45]. All experimental procedures were performed in accordance with the principles and guidelines adopted by NIH and approved by the Tulane IACUC. Transcriptomes from these experiments are available under study accession SRP093363 through NCBI’s Sequence Read Archive.

### Cell culture

Human colon cancer cells HT29, HCT116, and SW620 were obtained from the American Type Culture Collection (ATCC, Manassas, VA). HT29 and HCT116 cells were propagated in complete McCoy’s 5 A media (Sigma, St. Louis, MO) containing 10% fetal bovine serum (FBS) (Peak Serum, Wellington, CO). SW620 cells were grown in Leibovitz L-15 media (ATCC) containing 10% FBS. Mouse colon cancer CMT93 cells (ATCC) were propagated in DMEM (Sigma) containing 10% FBS. Human non-transformed colonic NCM460 cells were obtained from INCELL Corporation (INCELL, San Antonio, TX) and were grown in an M3Base medium (INCELL) containing 10% FBS. All colon cancer cell lines have been authenticated by ATCC through a short tandem repeat (STR) DNA profile and were tested for mycoplasma contamination. Cells were serum starved overnight prior to experimental procedures and were serum starved for three days to synchronize cells in the cell cycle prior to treatments.

### Colonospheres culture

A total of 5 × 10^6^ human colon cancer cells were plated in 24-well ultra-low attachment plates (S-bio, Hudson, NH) and propagated in stem cell media (DMEM-F12 with 5 mM HEPES) (ThermoFisher, Waltham, MA) supplemented with ITS liquid media complement (Sigma), bovine serum albumin (BSA, 4 mg/ml) (Sigma), l-glutamine (2 nM) (Sigma), epidermal growth factor (EGF, 20 ng/ml) (Invitrogen, Waltham, MA), and basic fibroblast growth factor (bFGF, 20 ng/ml) (Invitrogen) [46]. Colonospheres enriched with cancer stem cells were imaged using light microscopy and colon cancer stem cell populations were validated with the stem cell marker CD133.

### Reagents

The following reagents were utilized for our study: Oleic acid (OA) (300 µM) (Sigma), atglistatin (40 µM) (Cayman, Ann Arbor, MI), doxycycline (dox, 2 ug/mL) (Sigma), puromycin (250 ng/ml) (Sigma), bovine serum albumin (BSA, 4 mg/ml) (Sigma), l-glutamine (2 nM) (Sigma), epidermal growth factor (EGF, 20 ng/ml) (Invitrogen), basic fibroblast growth factor (bFGF, 20 ng/ml) (Invitrogen), 12-carbon chain saturated fatty acid analog with orange-red fluorescently labeled probe C_12_ (1 µM) (Invitrogen), and BrdU (10 µM) (Sigma). The following specific antibodies against protein were used: conjugated phycoerythrin (PE) CD133 (Biolegend, San Diego, CA), ATGL (Abcam, Waltham, MA), CD133 (Abcam), phosphorylated retinoblastoma (pRb Ser807/811) (Cell Signaling, Danvers, MA), total retinoblastoma (SantaCruz Biotech, Dallas, TX), MYC (Abcam), β-actin (Cell Signaling), BODIPY 493/503 (1 µg/mL) (Invitrogen), DAPI (1 µg/mL) (Invitrogen), and BrdU (Cell Signaling).

### Stable, inducible shRNA colon cancer cell clones

For generating stable short hairpin RNA (shRNA) knockdown cell lines, ATGL-specific shRNA (shATGL) and control shRNA were cloned into the TET-ON all in one LT3GEPIR vector using the following nucleotide shRNA guide sequences: 5′-TCTCAATCTGACATCTACGA-3′ (shATGL), 5′-TAGATAAGCATTATAATTCCTA-3′ (shCon) [47]. Sequence verified plasmids were transfected into HCT116 cells using lipofectamine 3000 according to the manufacturer’s instructions (Invitrogen). Stable clones were selected using puromycin (250 ng/ml) over several weeks. Knockdown of ATGL was induced by treatment of clones with doxycycline (dox) (2 ug/mL) for 48 h.

### siRNA

Human colon cancer cells were transfected with ATGL-specific pooled siRNA (MISSION esiRNA EHU003547) (Sigma) or equal amounts of negative-control scramble oligonucleotides using lipofectamine 3000 according to manufacturer’s instructions (Invitrogen). The efficiency of the knockdown validated by immunoblot was more than 60%.

### Flow cytometry

Human colon cancer cells, serum starved for three days followed by treatments, fixed (ethanol), permeabilized (Triton-x 100), and stained for DNA (Propidium Iodide) were used for flow cytometry analysis (Cytek Aurora, 4 laser system). The experiment was performed four independent times and data were analyzed using SpectroFlo and Modfit software. Flow cytometry analysis was also performed using conjugated phycoerythrin (PE) anti-human CD133 antibody (Biolegend, San Diego, CA) for the colon cancer stem cells population. The experiment was performed three independent times and data were acquired using BD FACS Aria III and results were analyzed by BD FACSDiva software.

### Protein extraction and immunoblotting

Protein extraction and immunoblot were performed as described previously [48]. The following specific antibodies against protein were used: ATGL (independent experiments more than ten times by different investigators) (Abcam), CD133 (Abcam), phosphorylated retinoblastoma (independent experiments three times by different investigators) (pRb Ser807/811) (Cell Signaling, Danvers, MA), total retinoblastoma (independent experiments three times) (Santa Cruz Biotech, Dallas, TX), MYC (1 time) (Abcam), and β-actin (Cell Signaling) (independent experiments more than 20 times by different investigators). Proteins were visualized with IRDye-conjugated secondary antibodies (LI-COR, Lincoln, NE) using the Odyssey infrared imaging system (LI-COR).

### BODIPY immunofluorescent staining

Experimental cells grown on coverslips were fixed with 3.7% paraformaldehyde and immunofluorescently stained for LDs as previously described [24]. Briefly, fixed monolayers were incubated with 1 µg/mL BODIPY 493/503 (Invitrogen) for 30 min (independent experiments three times by different investigators). Slides were washed, mounted with Prolong Gold antifade reagent containing DAPI (Invitrogen), and observed using fluorescent microscopy (Olympus DP80 with CellSens Dimension camera software).

### LDs dynamics assay

Human colonospheres were incubated (overnight) with a 12-carbon chain saturated fatty acid analog with orange-red fluorescently labeled probe C_12_ (1 µM) (Invitrogen). After washing (ten times with PBS), colonospheres were propagated in their media for 48 h and LDs were imaged using a fluorescent microscope (Olympus DP80 with CellSens Dimension camera software) (independent experiments three times by different investigators).

### BrdU staining

Human colon cancer cells, siRNA transfected and grown on coverslips, were treated with OA and with BrdU (10 µM) (Sigma) for the final 6 h. Cells were fixed with 3.7% paraformaldehyde and stained with anti-BrdU antibody (Cell Signaling) and visualized using the slide scanner Aperio CS2 (Leica) and Image Scope software (independent experiments four times). BrdU positive cells were counted as a percentage of total cells per high power field.

### Colony formation assay

Colon cancer cells (10^3^) were plated in a six-well plate. After 8 days, cells were washed and stained with 0.5% crystal violet following incubation with methanol (20 min) and quantified (OD570 nm) (independent experiments more than five times).

### Migration assay

Colon cancer cells (2.5 × 10^4^) were plated in the upper chamber of a 24-well transwell (Millipore, Burlington, MA) in serum-free media. Media containing a 10% FBS was added to the lower chamber of these transwells followed by treatment with atglistatin. After 24 h, the upper side of membranes from the transwell insert were scrubbed to remove non-migrated cells. Cells on the lower side of membranes were fixed in formalin and stained with crystal violet. Membranes were excised from the transwell insert and mounted on glass slides. The number of migrated cells were visualized by microscopy and counted per high power field (independent experiments two times).

Another approach utilized for assessing migration involved the disruption of confluent colon cancer cell monolayer using a p200 pipet tip and debris was removed to smooth the edges followed by treatments. After 24 h, the distances between migratory fronts (gap closure) were visualized and quantified using light microscopy (independent experiments three times by different investigators).

### RNA isolation and cDNA synthesis

Isolation of total RNA was accomplished using the miRNeasy kit (Zymo Research, Irvine, CA) according to the manufacturer’s instructions. RNA quality was determined by an Agilent Bioanalyzer (Agilent Technologies, Santa Clara, CA) and samples with RNA integrity numbers (RIN) >7 were used. For qPCR, RNA treated with DNase was reverse transcribed to cDNA with the qScript cDNA SuperMix synthesis system (Quantabio, Beverly, MA) according to the manufacturer’s protocol.

### qPCR

cDNA generated from human colon cancer cells and colonospheres was utilized for qPCR as previously described [45] (independent experiments three times by different investigators). The following primers were used for amplification of human cDNA: (hATGL-FOR 5′-AGTGACATCTGTCCGCAGG-3′, hATGL-REV 5′-CGGTTCAGGAGGCCGTT-3′, hATG2B-FOR 5′-GGTTCAAACCATACAGGCAGC-3′, hATG2B-REV 5′-AATGGTCTGAGCCGTGTCTG-3′, hPCK2-FOR 5′- GAAAACCCTGATTGGCCACG-3′, hPCK2-REV 5′-GATGCCCAGGATCAGCATGT-3′, hMYC-FOR 5′- TGGAAAACCAGCCTCCCG-3′, hMYC-REV 5′-TTCTCCTCCTCGTCGCAGTA-3′, hKLF6-FOR 5′-CTCCCACTGTGACAGGTGTT-3′, hKLF6-REV 5′-ACAGGATCCACCTCTCTGCT-3′, hPGAM1-FOR 5′- ATCTGCTAATCCCAGTCGGTG-3′, hPAGM1-REV 5′- CAAACTCATAGCCAGCATCTCG-3′, hSPTLC2-FOR 5′- ATGGCACCAGCCTTGGTAAAG-3′, hSPTLC2-REV 5′-AGCCCATCTCTTTCAGGCG-3′, hIGFBP1-FOR 5′-CATCCTTTGGGACGCCATCA-3′, hIGFBP1-REV 5′-ATGGATGTCTCACACTGTCTGC-3′, hMUC2-FOR 5′-GGAAGAACGATGTGTGTGCC-3′, hMUC2-REV 5′-TGTTATGGACGCAAGGGCAT-3′, hABCC3-FOR 5′-CAGGCCAGTGTGTCTCTGAAA-3′, hABCC3-REV 5′- GTATGGTGATGGCATAGCCTGG-3′, hDSG4-FOR 5′- CATCCTGCTACTGATTTTGGCTC-3′, hDSG4-REV 5′-GGCGTAGGCGCTAAAAAGC-3′, hPAPPA2-FOR 5′-TAAGGAGCAAAACACTTGGAACC-3′, hPAPPA2-REV 5′- TGCTTGGGGTTGTTGGTTGT-3′, hSEC24D-FOR 5′-GGAGCGGGAACAGACTTCTT-3′, hSEC24D-FOR 5′-GGAGGTGTAGCCACGTAACC-3′, hACACA-FOR 5′-GGTGAAGAGGGTGCGTTTCA-3′, hACACA-REV 5′-CACTTCCAAAAAGACCTAGCCC-3′, hSQSTM1-FOR 5′- CATTGCGGAGCCTCATCTCC-3′, hSQSTM1-REV 5′-TCCCCGTCCTCATCCTTTCT-3′, hELF3-FOR 5′-CCGAGCAAGAGCGTAGCC-3′, hELF3-REV 5′-CAGTCCAGAACCTGCGTCTT-3′, hABCG1-FOR 5′-CGCTTTCTCGGTCGGCA-3′, hABCG1-REV 5′-TGCCCGTCTCCCTGTATCC-3′). To determine the relative levels of mRNA, the comparative Ct method was employed using Actin and GAPDH as a housekeeping control. The C1000 Thermal Cycler system (Bio-Rad, Hercules, CA) and PerfeCTa SYBR Green FastMix (Quantabio) were used to quantify cDNA.

### RNA sequencing and differential expression testing

RNA sequencing (RNAseq) of experimental colon cancer cells and colonospheres (*n* = 3 for each group) was accomplished as described previously [45, 49]. Sequencing data along with the study design will be submitted to NCBI’s Sequence Read Archive and will be publicly available following the publication of the manuscript.

### Transcriptome and pathway analysis

RNAseq data analysis was performed using Ingenuity Pathway Analysis (IPA) (Qiagen, Germantown, MD). Differentially expressed genes (DEGs) entered into IPA met an expression threshold of >|1.5| -fold change with respect to control and a false discovery rate (FDR) <0.05. Clustered heatmaps were generated reflecting z-scaled transcripts per million (TPM) values for the top genes across all samples using a Python data visualization package (Seaborn). Expression of select transcripts was based on data generated by the TCGA research network, (https://cistrome.shinyapps.io/timer) [50].

### Histological analysis

Processing and immunohistostaining for human colon cancer and matched normal tissue were performed by The Pathology Core Laboratory at Tulane University Health Sciences Center (http://medicine.tulane.edu/departments/pathologylaboratorymedicine/research/histology-laboratory) as described previously [45, 49]. Heat-induced epitope retrieval was performed on tissue sections using Decloaker solution (BioCare Medical, Pacheco, CA) and cooked in an Oster steamer for 40 min. Sections were blocked using Block M (BioCare Medical) followed by incubation with the following antibody: ATGL (1 h) (Abcam). Following washing, tissue sections were incubated with Rabbit HRP-Polymer secondary (BioCare Medical); sections were then washed and treated with Betazoid DAB chromogen (Biocare Medical) followed by counterstaining with Cat hematoxylin. Slides were dried in the oven, placed in xylene, and coverslipped (Acrymount) (Statlab, McKinney, TX). Images were obtained using the slide scanner Aperio CS2 (Leica) and Image Scope software. Images were quantified utilizing ImageJ by performing spectrum deconvolution for separation of DAB (diaminobenzidine) color spectra. The DAB image was then analyzed pixel by pixel for immunohistochemistry scoring analysis.

### Statistical analysis

To ensure statistical significance and data reproducibility, all experiments included a minimum of three samples per group and were repeated independently by the same or different researchers. All data are means ± SE for a series of experiments. Data meet the normal distribution and statistical analysis was performed by Student’s unpaired *t*-test or one-way analysis of variance (ANOVA) and Student Newman–Keuls post-test using Graph Pad Instat 3 software (Graph Pad Software). A *p* value <0.05 was considered significant. Investigators were blinded to group allocation during the experiment. After assessing the outcomes, investigators traced back the group (samples) allocation for assembly of data and generated figures.

## Results

### ATGL is increased in human colonic tumors and augmented by obesity

**Fig. 1:**
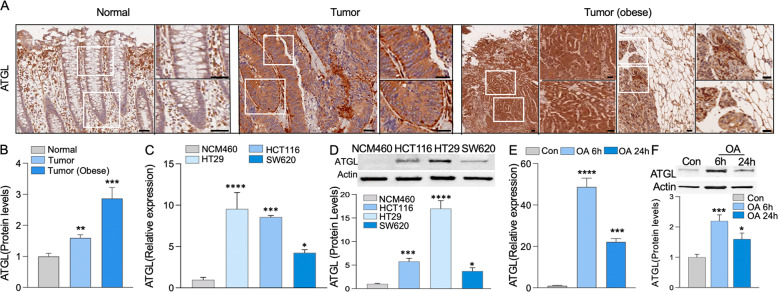
ATGL level is increased in human colon cancer tissue and augmented by obesity. **A**, **B** Human colon cancer tissue and matched control from local patients, including obese individuals (tumor with adjacent adipose tissue from obese colon cancer patients, BMI: 23–40 kg/m^2^), were immunohistostained for ATGL. Images of immunohistostainings including focus insets represented colonic crypts and tumors. Quantification of immunohistostained ATGL of the tissue was done by ImageJ, performing spectrum deconvolution for separation of DAB (diaminobenzidine) color spectra. The DAB image was then analyzed pixel by pixel for immunohistochemistry scoring analysis. Graph represents immunohistostaining quantification (ImageJ, *n* = 10 patients, **p < 0.01, ***p < 0.001, scale bar 40 µm). **C**, **D** Increased ATGL mRNA and protein levels in transformed colon cancer cells (HT29, HCT116, and SW620) relative to non-transformed (NCM460) colonic cells. Actin was used as a loading control. Graphs represent ATGL levels (mRNA) and ATGL densitometric quantification (protein) (*n* = 3, *p < 0.05, ***p < 0.001, ****p < 0.0001). **E**, **F** In HT29 cells, OA augmented ATGL levels (mRNA, protein) at 6 and 24 h relative to control. Actin is used as a loading control. Graph represents ATGL levels (mRNA) and ATGL densitometric quantification (protein) (*n* = 3, *p < 0.05, ***p < 0.001, ****p < 0.0001).

Since elevated LDs levels in colonic tumor tissue are critical for colon cancer cells growth [21–24], it is important to understand how they provide the fuel needed for cancer progression. Therefore, ATGL, the LD-associated lipase responsible for LDs utilization, was assessed by immunohistostaining in human colonic tumor tissue obtained from local patients. In normal tissue, ATGL was localized in the cytosol of colonic cells, along the crypt, and in the surrounding cells. In tumor tissue, cytosolic ATGL levels were significantly increased compared to matched controls. The increase in ATGL levels was further augmented in colonic tumors of obese individuals (Fig. 1A, B).

Furthermore, in human colon cancer cells (CCC), HT29, HCT116, and SW620, ATGL levels (mRNA and protein) were elevated relative to non-transformed colonic NCM460 cells (Fig. 1C, D). Among these cells, HT29, which has the highest capacity for LDs accumulation [22, 25], showed augmented ATGL levels relative to HCT116 and SW620 cells. The obesity mediator, oleic acid (OA), further increased ATGL levels in HT29 cells as shown by mRNA and protein assessment (Fig. 1E, F). Specifically, OA stimulated an increase in ATGL levels at 6 h that was higher compared to 24 h. This suggests that at 24 h, a negative regulatory feedback mechanism for LDs accumulation (as we previously described [24, 26]) may also involve ATGL-mediated LDs utilization. Together these data revealed increased ATGL levels in human colonic tumors and CCC, which were augmented by obesity and an obesity mediator, OA.

### Increased ATGL facilitates colon cancer cell migration and growth

Next, we determined the role of increased ATGL in colon cancer progression. We utilized atglistatin, a specific pharmacological inhibitor [51], or siRNA to examine if targeting ATGL in CCC prevents LDs utilization. In human colonic non-transformed NCM460 cells, human CCC (HT29), and mouse CCC (CMT93), atglistatin led to a significant increase in LDs levels following OA treatment (Supplemental S1A). Similarly, in human HT29 with silenced ATGL (siRNA), OA treatment further increased LDs levels (Supplemental S1B). These data support that effective inhibition of ATGL in CCC, either by atglistatin or small interfering RNA, leads to blockade of LDs utilization.

**Fig. 2:**
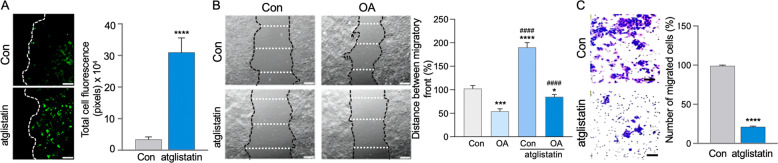
The obesity mediator stimulated colon cancer cell migration is attenuated by ATGL inhibition. **A** In HT29 cells following confluency disruption, LDs accumulate in cells close to the migratory front and were further elevated by ATGL inhibition (atglistatin). LDs were visualized by staining with BODIPY (green). Graph represents total cell fluorescence quantification (pixels) for BODIPY (*n* = 3, ****p < 0.0001, scale bar 40 µm). **B** Confluency disruption of HT29 monolayers revealed increased gap closure between migratory fronts following OA treatment which was impeded by atglistatin. Graph represents quantification of distance between migratory fronts (*n* = 3, *p < 0.05, ***p < 0.001, ****p < 0.0001 compared to Con, ^####^*p* < 0.0001 compared to OA, scale bar 40 µm). **C** Migration of HCT116 cells from the upper to the lower chamber of transwell was attenuated by ATGL inhibition (atglistatin). Graph represents percentage of migrated cells (*n* = 3, ****p < 0.0001, scale bar 40 µm).

Human CCC, stimulated to migrate following confluency disruption, had increased LDs accumulation in cells close to the migratory front that was further elevated with ATGL inhibition (atglistatin) (Fig. 2A). Further, OA-facilitated HT29 cells migration after confluency disruption, as shown by increased gap closure between migratory fronts, was diminished by atglistatin (Fig. 2B). Similarly, utilizing another approach, atglistatin significantly reduced migration of HCT116 from the upper to lower chamber of the transwell (Fig. 2C). These data support the important role of ATGL-mediated LDs utilization in the migration of CCC.

**Fig. 3:**
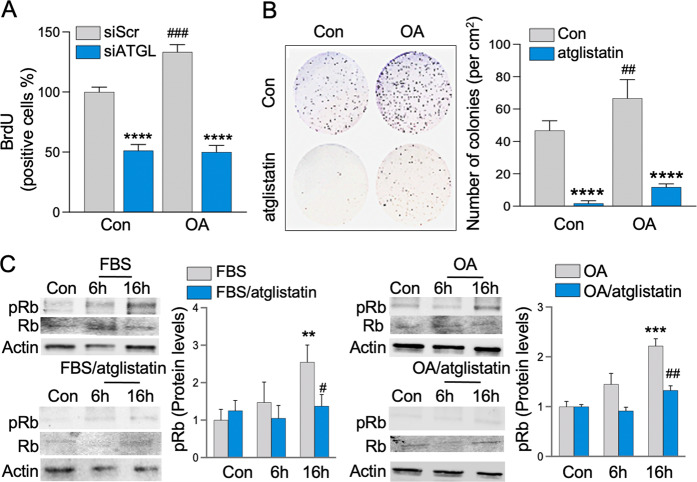
The obesity mediator stimulated colon cancer cells progression is attenuated by ATGL inhibition through cell cycle arrest. **A** Silenced ATGL in HT29 cells attenuated OA stimulated DNA synthesis. HT29 cells transfected with siRNA for ATGL (siATGL) and control with scrambled siRNA (siScr), propagated on coverslips (48 h) were treated with BrdU and stimulated with OA for the final 6 h. Coverslips were stained with BrdU antibody and BrdU positive cells were counted and presented as percentage of total cells per high power field relative to control (BrdU staining, *n* = 3, ****p < 0.0001 compared to Con siScr or OA siScr, ^###^*p* < 0.001 compared to Con siScr). **B** ATGL inhibition with atglistatin lowered colon cancer cells colonies. HCT116 cells, grown on six-wells plate with atglistatin, with and without OA and colony number was determined 8 days later. Graph represents number of quantified colonies per cm^2^ relative to control (*n* = 4 and 8, ****p < 0.0001 compared to Con or OA, ^##^*p* < 0.01 compared to Con). **C** HT29 cells were serum starved for three days followed by treatment with FBS and OA (6 and 16 h) with and without inhibition of ATGL (atglistatin). Increased phosphorylation of Rb by FBS and OA at Ser807/811 was attenuated by atglistatin. Total Rb and actin were used as a control. Graph represents pRb densitometric quantification (*n* = 3, **p < 0.01, ***p < 0.001 compared to Con, ^#^*p* < 0.05, ^##^*p* < 0.01 compared to FBS and OA respectively at 16 h).

Moreover, in HT29 cells with silenced ATGL (siRNA), OA-stimulated BrdU incorporation into newly synthesized DNA (6 h) was significantly attenuated (Fig. 3A). In another approach, the presence of atglistatin led to significantly less HCT116 colonies (8 days), including those stimulated with OA (Fig. 3B). Consequently, serum starved HT29 (for cell cycle synchronization in G0 phase) [50] stimulated with FBS or OA showed phosphorylation of retinoblastoma protein (Rb) at S807/811 sites, which are specific for cell cycle arrest [52]. In the presence of atglistatin, Rb phosphorylation was significantly reduced (Fig. 3C). Further, FACS analysis demonstrated that ATGL (24 h) inhibition (atglistatin) retained HT29 cells in the G1 phase and thereby diminished their presence in the G2 phase of the cell cycle (Supplemental S2A, B). These findings demonstrate that targeting ATGL-mediated LDs utilization effectively attenuates CCC growth, in part, through cell cycle arrest.

### Inhibition of ATGL-targeted pathways that drive tumorigenesis, including those facilitated by high-fat-diet obesity

**Fig. 4:**
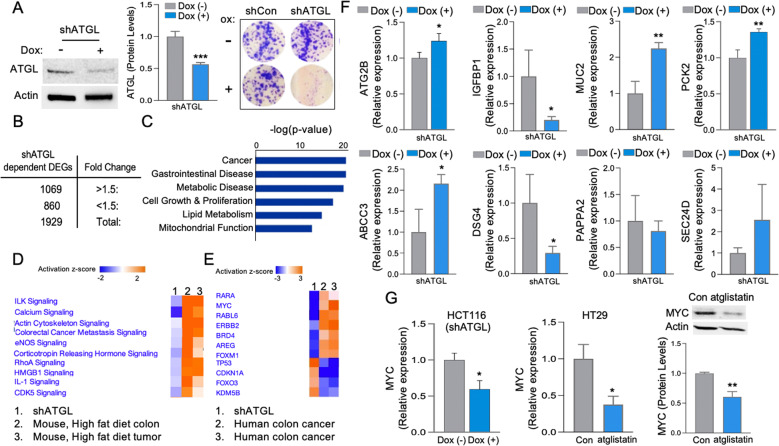
Increased ATGL mediates transcriptional remodeling linked to colonic tumorigenesis, driven by obesity. **A** HCT116 cells with stable transfected shATGL clone (dox-inducible) showed reduced ATGL levels and colony formation (8 days). Actin as a loading control. Graph represents ATGL densitometric quantification (Colony formation assay, *n* = 6, ***p < 0.001). **B** Differentially expressed genes (DEGs) from HCT116 clone with shATGL (dox-induced knockdown for 2 days) relative to control (*n* = 3 for each group, FC > |1.5| , FDR < 0.05). **C** Top diseases affected by shATGL knockdown relative to control (*p* < 0.05, IPA). **D** Top canonical pathways altered in high-fat-diet obese mice colon and tumors were targeted with ATGL knockdown (shATGL) (*p* < 0.05, IPA). DEGs representing colon and colonic tumors of high-fat-diet obese mice relative to colon of control mice (*n* = 3 for each group, FC > I1.5I, FDR < 0.05). **E** Upstream regulators associated with DEGs representing human colon cancer tissue relative to normal colon (GSE20916, GSE62322, FC > I1.5I, FDR < 0.05) were targeted by ATGL knockdown (shATGL) (*p* < 0.05, IPA). **F** Validation of altered ATG2B, IGFPBP1, MUC2, PCK2, ABCC3, DSG4, PAPPA2, and SEC24D transcripts in shATGL HCT116 clone (dox-induced knockdown for 2 days) (qPCR, *n* = 3, *p < 0.05, **p < 0.01). **G** Inhibition of ATGL led to attenuated MYC levels. MYC level following ATGL inhibition in HCT116 clones (shATGL, dox-induce for 2 days) and HT29 (atglistatin for 1 day) were assessed by qPCR and IB (actin as a loading control). Graphs represents MYC levels (qPCR) and protein densitometric quantification (protein) (*n* = 2, *p < 0.05, **p < 0.01).

Since the above findings revealed that ATGL plays an important role in colon cancer progression, we further investigated systemic transcriptional changes that are dependent on ATGL and also associated with these processes. To determine if targeting ATGL would have an inhibitory effect on heterogenic colon cancer cell populations, we utilized two CCC lines, HCT116 and HT29, with different approaches of ATGL targeting: shRNA and atglistatin. Selected clones of stably transfected HCT116 cells with shATGL under doxycycline showed substantially lowered ATGL and colonies (8 days) (Fig. 4A), similar to what we presented above (Fig. 3B) when ATGL was targeted by atglistatin in HT29 cells. Transcriptomic analysis (RNAseq) of HCT116 clones with shATGL, following 2 days of doxycycline, showed a significant number of differentially expressed genes (DEGs), among which 1069 were increased and 860 were decreased (Fig. 4B) (>|1.5| -fold change, FDR <0.05). The top fifty of these shATGL-dependent DEGs are presented as a table in Supplemental S3. Diseases and pathway analysis of these DEGs revealed associations with cancer, gastrointestinal and metabolic diseases, as well as biological processes related to metabolism (lipids and mitochondria) and growth (Fig. 4C, IPA). Next, we assessed if high-fat-diet obesity mediating pathways in mouse colon and colonic tumors [45] may be targeted by ATGL inhibition. Specifically, activated pathways associated with transcriptomes of high-fat-diet obese mouse colon and colonic tumor (relative to tissue from non-obese mice) were attenuated with ATGL inhibition (from shATGL clone relative to control) (Fig. 4D, IPA). Additionally, shATGL-dependent DEGs were compared to publicly available transcriptomes from human colon cancer patients’ affected tissue (GSE20916 and GSE62322). We found that inhibition of ATGL abrogated critical drivers of colon cancer progression including RARA, MYC, ERBB2, AREG, and FOXM1, while promoting those with tumor-suppressive function such as FOXO3, TP53, and CDKN1A (Fig. 4E, IPA). Selected shATGL-dependent DEGs, were validated for altered levels in HCT116 shATGL clones (Fig. 4F) and HT29 cells treated with atglistatin (Supplemental S4). This included validation of autophagy-related 2B (ATG2B), insulin-like growth factor-binding protein 1 (IGFBP1), mucin 2 (MUC2), phosphoenolpyruvate carboxykinase 2 (PCK2), canalicular multispecific organic anion transporter 2 (ABCC3), and desmoglein 4 (DSG4) with significant alterations (*p* < 0.05) or similar trends such as protein transport protein 24D (SEC24D) and pappalysin 2 (PAPPA2). These DEGs, significantly altered in human colon cancer tissue (TCGA) (Supplemental S4), are ones with both emerging roles (SEC24D, ABCC3, ATGL2B, and PCK2) and established roles (MYC and MUC2) in colonic tumorigenesis. Moreover, the above data (Fig. 4E) revealed that targeting ATGL abrogated MYC, the critical oncogene that also plays a role in metabolism [53, 54]. We further validated lowered MYC level (mRNA and protein) in HCT116 (shATGL) and HT29 (atglistatin) cells (*p* < 0.05) (Fig. 4G). Together, these data revealed that inhibition of ATGL targets metabolic and tumorigenic pathways in addition to multiple genes involved in diverse cellular functions that drive human colon cancer progression, especially when facilitated by high-fat-diet obesity.

### ATGL-mediated LDs utilization in colonospheres is critical for growth

**Fig. 5:**
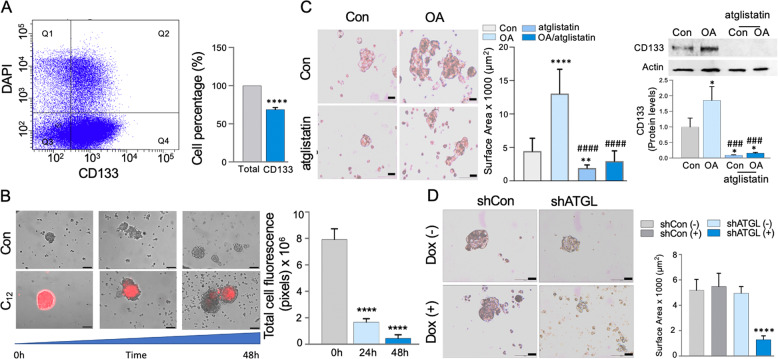
The obesity mediator stimulated growth of human colonospheres is attenuated by ATGL inhibition. **A** HT29 colonospheres were enriched in colon cancer stem CD133 positive cells shown in fourth quadrant (Q4). Graph represent percentage of CD133 positive cells (FACS, *n* = 3, ****p < 0.0001). **B** HT29 colonospheres were incubated with fluorescently labeled 12-carbon chain fatty acid analog (C_12_) followed by next day C_12_ removal from media and extensive washing. In these enlarged colonospheres during 48 h growth intracellular LDs-C_12_ (red) become substantially diminished. Graph represents total cell fluorescence quantification (pixels) for C_12_ (*n* = 3, ****p < 0.0001, scale bar 100 µm). **C** OA induced HT29 colonospheres growth shown by imaging and increased surface area calculation was attenuated with atglistatin (*n* = 9–22 colonospheres, **p < 0.01, ****p < 0.0001 compared to Con, ^####^*p* < 0.0001 compared to OA, scale bar 20 µm). ATGL inhibition further lowered OA-stimulated CD133 positive cancer stem cells. Actin as a loading control. Graph represents CD133 densitometric quantification (protein) (*n* = 3, *p < 0.05 compared to Con, ^###^*p* < 0.001 compared to OA). **D** HCT116 clones with knockdown ATGL (shATGL, dox- inducible) propagated as colonospheres showed decreased growth relative to control. Representative image and surface area quantification (*n* = 7–16 colonospheres ****p < 0.0001, scale bar 20 µm).

Resistance to therapy and cancer recurrence in colon cancer patients, both prevalent in obese individuals, are mainly due to the heterogenic nature and regenerative potential of colon cancer stem cells (CCSC) [4, 5, 55–57]. Since emerging studies showed that CCC response to therapies may depend on LDs [25], we hypothesize that LDs dynamics in CCSC could be a shared characteristic among heterogenic CCSC populations and thus present an effective target. Therefore, colonospheres, derived from HT29 cells, enriched for CCSC (CD133 positive, Fig. 5A) were propagated in the presence of fluorescently labeled 12-carbon chain fatty acid analog (C_12_) that is shown to be stored in LDs [22, 58]. After removal of C_12_ from media, we found a significant decrease in intracellular LDs-C_12_ (red) during the growth of colonospheres (48 h) (Fig. 5B). These data suggest that CCSC accumulated and subsequently utilized LDs. Further, OA-stimulated growth of colonospheres, as represented by their increased surface area and CD133 marker, was attenuated by inhibition of ATGL with atglistatin (Fig. 5C). Similar growth inhibition was seen in colonospheres derived from HTC116 clones with shATGL (Fig. 5D). These data reveal that ATGL-mediated LDs utilization was important for the growth of colonospheres enriched for CCSC.

### Inhibition of ATGL-targeted pathways that drive tumorigenesis in colon cancer stem cells

**Fig. 6:**
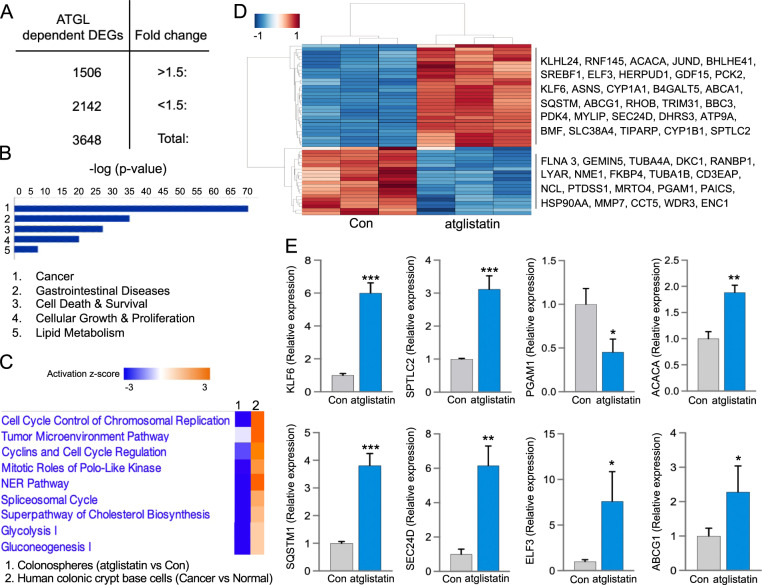
ATGL-dependent transcriptional remodeling in colonospheres. **A** Differentially expressed genes (DEGs) from atglistatin treated HT29 colonospheres relative to control (*n* = 3 for each group, FC > |1.5| , FDR <0.05). **B** Top diseases and pathways affected by ATGL inhibition in colonospheres (*p* < 0.05, IPA). **C** Altered pathways in human colonic tumor crypt base cells (enriched in cancer stem cells) from colon cancer patients compared to normal crypts (GSE20916) were targeted by ATGL inhibition in colonospheres (*p* < 0.05, IPA) **D** Top fifty ATGL-dependent DEGs shown as a clustered heatmap reflecting z-scaled transcripts per million values for the genes in colonospheres (*n* = 3, *p* < 0.05, Seaborn). **E** Validation of select ATGL-dependent KLF6, SPTLC2, PGAM1, ACACA, SQSTM1, SEC24D, ELF3, and ABCG1 transcripts in HT29 colonospheres (qPCR, *n* = 3, *p < 0.05, **p < 0.01, ***p < 0.001).

We further identified in colonospheres ATGL-dependent transcriptional alterations linked to tumorigenesis. Initial data showed that colonospheres (derived from HT29), relative to adherent HT29 cells, have 1265 differentially expressed genes (>|1.5| -fold change, FDR < 0.05) (Supplemental S5A), suggesting their distinctive transcriptional profile. The top diseases and functions associated with these DEGs were cancer, gastrointestinal diseases, metabolic processes, and growth (Supplemental S5B, IPA). To further determine if these DEGs are related to human CCSC, we analyzed publicly available transcriptomes from colonic tumor crypt base (enriched with CCSC) relative to control crypt base obtained from colon cancer patients (GSE20916). Pathways analysis of these DEGs showed strong similarity between colonospheres (relative to adherent) and human colonic tumor crypt base (relative to normal) obtained from colon cancer patients (Supplemental S5C, IPA). Furthermore, we examined if inhibition of ATGL in colonospheres would target pathways driving colonic tumorigenesis. We found that colonospheres with inhibited ATGL (atglistatin) relative to control have 1506 upregulated and 2142 downregulated DEGs (>|1.5| -fold change, FDR < 0.05) (Fig. 6A). These DEGs were linked to cancer and intestinal disease, as well as dysregulation of metabolism and growth (Fig. 6B, IPA). Also, a comparison of DEGs from colonospheres with blocked ATGL (atglistatin) to colon cancer cells (shATGL clone) demonstrated shared and unique pathways alterations (Supplemental S6), suggesting a characteristic transcriptional profile for colonospheres. Additionally, to determine whether targeting ATGL would effectively suppress pathways in human CCSC, we compared these DEGs representing colonospheres treated with atglistatin to DEGs from the publicly available transcriptome of human colonic tumor crypt base cells (relative to control) from cancer patients (GSE20916). We found that blockade of ATGL-targeted pathways altered in human colonic tumors crypt base (Fig. 6C, IPA), suggesting that ATGL blockade may be effective for inhibiting the heterogenic human CCSC [59] from the colonic tumor crypt base. Several of these targeted pathways were associated with growth (cell cycle) and metabolism (lipids and glycolysis). The top fifty of these DEGs specific to ATGL-targeted colonospheres (Supplemental S7, table), presented by heatmap, were associated with stem cell function (Lgr5, Ascl2, Olfm4), mitochondrial function (PTDSS1), and lipid metabolism (CYP1A1, PCK2, and SREBF1) (Fig. 6D). Selected DEGs were validated for ATGL-mediated expression in colonospheres from two cell lines, HT29 (Fig. 6E) and HCT116 (Supplemental S8) treated with atglistatin. The validated DEGs with significant (*p* < 0.05) or trending alteration in colonospheres from both cell lines included: kruppel like factor 6 (KLF6), serine palmitoyltransferase long chain base subunit 2 (SPTLC2), phosphoglycerate mutase 1 (PGAM1), acetyl-CoA carboxylase alpha (ACACA), sequestosome 1 (SQSTM1), protein transport protein 24D (SEC24D), E74 Like ETS Transcription Factor 3 (ELF3), and ATP Binding Cassette Subfamily G Member 1 (ABCG1). Their altered levels were also found in colon cancer patients (TCGA) (Supplemental S8). Further, among validated DEGs were those with established roles in colon cancer and CCSC (KLF6 and SQSTM1) and those with emerging roles (SPTLC2, SEC24D, and ACACA). Together, this data showed that ATGL inhibition targets multiple pathways and genes in colonospheres driving colonic tumorigenesis and revealed a role for LDs-ATGL in colon cancer stem cells.

## Discussion

Obesity increases the risk and progression of colon cancer, patients’ resistance to therapy, and disease recurrence [3–6, 10, 56]. Here, we demonstrated that obesity reinforces colonic tumorigenesis, in part, through enhanced ATGL-mediated LDs utilization in colon cancer cells and colon cancer stem cells. Blockade of ATGL targets metabolic and growth pathways that drive colonic tumorigenesis, especially when facilitated by obesity. These findings provide a platform to explore LD-ATGL-mediated mechanisms involved in colonic tumorigenesis augmented with obesity.

We showed that, in colon cancer tissue and colon cancer cells, increased ATGL levels, important for growth, are augmented by obesity and an obesity mediator. Recent findings in renal and colon cancer revealed that obesity stimulates tumor cells to adopt lipid-based metabolism while infiltrated immune cells adapt to glucose [60]. As increased LDs emerge as a hallmark of cancer progression [15, 16], elevated ATGL-mediated LDs utilization in colon cancer cells may be critical in driving tumorigenesis. Increased ATGL in pancreatic cancer correlates with the accumulation of adipose tissue and contributes to tumor growth [41]. Similarly, aggressive breast cancer is linked to an excess in surrounding adipocytes and increased ATGL-LDs in tumor cells [40, 61]. Moreover, upregulated ATGL in immune cells facilitates their activity and production of inflammatory mediators [62–64], which may exacerbate tumorigenic microenvironment. Our findings establish that in colon cancer cells, ATGL-mediated LDs utilization could be critical in driving colonic tumorigenesis especially in tumors driven by obesity.

This study further demonstrated the critical role of ATGL in the propagation of human colon cancer cells and colonospheres (enriched in colon cancer stem cells) as well as their obesity-stimulated growth. Targeting ATGL-mediated LDs utilization in colon cancer cells led to cell cycle arrest effectively attenuating their growth facilitated by an obesity mediator. Emerging findings revealed that obesity promotes the expansion of cancer stem cells in breast and cervical tumors [65, 66]. In mice, obesity mediated by high-fat-diet propagates the population of the colonic Lgr5+ stem cell by stimulating their growth and reducing apoptosis [55, 57]. Recent findings from lung and colonic tumors suggest a possible involvement of LDs in cancer stem cells growth and spheroid formation capacity [29, 67], the mechanisms of which are not well understood. We speculate that colon cancer patients’ resistance to therapy and tumor recurrence are driven by elevated LDs accumulation and utilization in the heterogenic stem cancer cell population. Thus, it is plausible that blockade of LD-ATGL is a promising approach for targeting, not only cancer cells but also the heterogenic cancer stem cell population and for effective response to treatments in colon cancer patients.

We identified ATGL-mediated systemic transcriptional remodeling in human colon cancer cells and colonospheres associated with cancer growth and metabolism. Emerging findings revealed the importance of metabolic processes associated with cancer stem cells [7, 27], the mechanisms of which are not well understood. Several ATGL-dependent pathways and differentially expressed genes in colon cancer cells and colonospheres are involved in metabolism (lipids, mitochondria, and glucose), tumorigenesis, cell adhesion, and transport. Aberrant levels and mutations of ATG2B, which is involved in autophagosome formation and LDs morphology [68], are found in several cancers [69, 70]. Our findings revealed that significantly lowered ATG2B levels in human colon cancer may be recovered by ATGL blockade. Further, PCK2 (cytosolic and mitochondrial isoforms), which is involved in the citric acid cycle (TCA) [71], have increased expression associated with enhanced growth of lung and prostate cancer [72, 73]. In colon cancer, the mitochondrial isoform of PCK2 has lower expression [74] and our findings suggested that targeting ATGL recovers PCK2 levels. Moreover, SPTLC2, critical for sphingolipid synthesis [75], is also ATGL-dependent in colonospheres. The role of SPTLC2 in cancer stem cells is unexamined, but its lowered expression in renal cell carcinoma leads to poor patient survival [76]. Another ATGL-dependent gene in colonospheres is PGAM1, which is involved in glycolysis and is highly expressed in colonic tumors thereby promoting their growth [77, 78]. One study showed that PGAM1 is elevated in breast cancer stem cells [79], but its role in colon cancer stem cells is not understood. These ATGL-dependent genes involved in the metabolism of lipids, mitochondria, and glucose suggest a cross-talk between metabolic regulators in colon cancer cells and colon cancer stem cells. Moreover, we showed that inhibition of ATGL lowers oncogenic MYC and increases tumor suppressor FOXO3. We have previously demonstrated that obesity-mediated by high-fat diet and deficiency in FOXO3 leads to LDs accumulation and activates MYC in mouse colon [45, 49]. Thus, it is plausible that the FOXO3 and LDs regulatory network, facilitated by obesity, may involve ATGL and MYC. Together, we have identified metabolic and tumorigenic genes driving colon cancer progression that may be targeted by ATGL inhibition.

Colon cancer incidents have been consistently increasing, especially among young adults, mainly due to obesity [3–6, 10, 56], posing an urgent, unmet demand to understand the mechanisms of obesity-mediated colonic tumorigenesis. Furthermore, the complexity of metabolic reprogramming, including LDs dynamics within cancer cells, emerged as an important hallmark of cancer progression [15, 16]. This metabolic reprogramming cross-talk with mechanisms driving tumorigenesis [15, 16] poses the critical question as to whether obesity and obesity mediators may highjack some of these pathways to promote colon cancer. Here, we demonstrated that one of the mechanisms through which obesity may reinforce colon cancer progression involves ATGL-mediated utilization of elevated LDs in colon cancer cells and colon cancer stem cells. This further provides a novel understanding of the interplay between metabolic and tumorigenic reprogramming, which will lead us to establish a platform for new diagnostic and therapeutic approaches in colon cancer, especially in obese patients.

## Supplementary information

Supplemental

## Data Availability

The datasets generated during the current study are available via NCBI GEO - accession number GSE195994.
