# Correction: Elevated ATGL in colon cancer cells and cancer stem cells promotes metabolic and tumorigenic reprogramming reinforced by obesity

**DOI:** 10.1038/s41389-022-00388-5

**Published:** 2022-03-15

**Authors:** Rida Iftikhar, Harrison M. Penrose, Angelle N. King, Joshua S. Samudre, Morgan E. Collins, Alifiani B. Hartono, Sean B. Lee, Frank Lau, Melody Baddoo, Erik F. Flemington, Susan E. Crawford, Suzana D. Savkovic

**Affiliations:** 1 Department of Pathology and Laboratory Medicine, Tulane University School of Medicine, New Orleans, LA USA; 2 Department of Surgery, Louisiana State University Health Sciences Center, New Orleans, LA USA; 3 NorthShore University Research Institute, Affiliate of University of Chicago Pritzker School of Medicine, Evanston, IL USA

**Keywords:** Cancer, Stem cells

Correction to: *Oncogenesis* (2021) 10:1–10 10.1038/s41389-021-00373-4, published: 29 November 2021

This article has been corrected to add a data availability statement:

**Data availability**

The datasets generated during the current study are available via NCBI GEO - accession number GSE195994.

The original article has been corrected.

