# Supplementary material for: Elevated ATGL in colon cancer cells and cancer stem cells promotes metabolic and tumorigenic reprogramming reinforced by obesity
Source: Oncogenesis. 2021 Nov 29;10(11):82. doi: 10.1038/s41389-021-00373-4 (PMC8630180; doi:10.1038/s41389-021-00373-4)
Supplement: Supplementary file 1 — Supplemental [file 41389_2021_373_MOESM1_ESM.pdf]

Supplemental S1

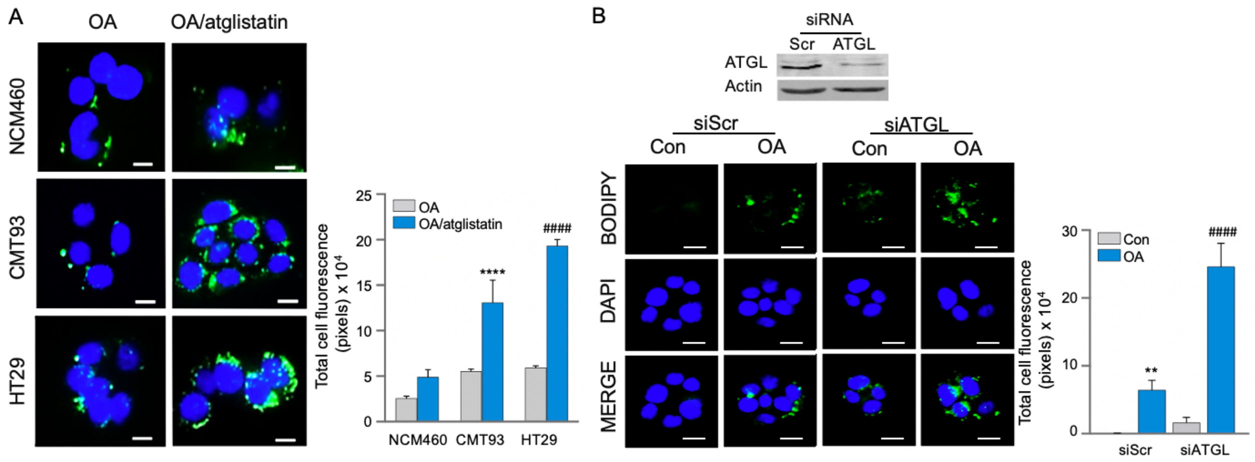

Supplemental S1. ATGL inhibition further augments OA stimulated LDs accumulation in colon cancer cells. A) OA stimulated LDs accumulation in human colonic non-transformed NCM460 cells, human colon cancer HT29 cells, and mouse colon cancer CMT93 cells was significantly augmented by pharmacologic inhibition of ATGL (atglistatin). LDs were visualized by staining with BODIPY (green). Graph represents total cell fluorescence quantification (pixels) for BODIPY (n=3, ****$p<0.0001$ compared to OA treated CMT93 cells, $^{####}p<0.0001$ compared to OA treated HT29 cells, scale bar 5μm). B) Silenced ATGL (siRNA) in HT29 cells significantly augmented OA induced LDs accumulation. LDs were visualized by staining with BODIPY (green). Graph represents total cell fluorescence quantification (pixels) for BODIPY (n=3, **$p<0.01$ compared to Con siScr, $^{####}p<0.0001$ compared to Con siATGL, scale bar 5μm).

Supplemental S2

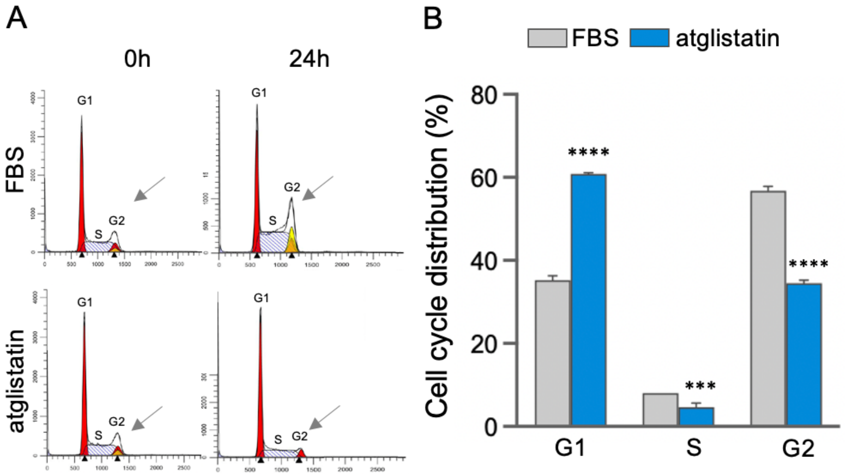

Supplemental S2. ATGL inhibition attenuates progression of colon cancer cells in cell cycle. A) FACS analysis of serum starved (for 3 days) colon cancer HT29 cells stimulated with FBS (24h) showed increased number of cells in G2 phase (grey arrow). In presence of ATGL inhibitor atglistatin, progression in G2 phase was attenuated and cells were retained in G1 phase of cell cycle (FACS, n=3). B) Graph representing percentage of cell cycle distribution following treatment with FBS and atglistatin. At 24h, ATGL inhibition promotes retention of colon cancer cells in G1 phase of cell cycle (n=3, ***$p<0.001$, ****$p<0.0001$).

Supplemental S3: Top fifty differentially expressed genes (DEGs) in human colon cancer cells with ATGL knockdown (HCT116-shATGL clones) relative to control (n=3, FC ≥|1.5|, p<0.05).

	Gene	Gene name	FC	Adj.P.Val
1	GNRHR2P1	GNRHR2 pseudogene 1	82.1	9.1E-13
2	KLHDC7B	Kelch domain containing 7B	60.7	4.8E-11
3	AMIGO3	Adhesion molecule with Ig like domain 3	53.2	2.1E-04
4	EIF4EP2	Eukaryotic translation initiation factor 4E pseudogene 2	52.9	2.5E-09
5	RAC1P2	Rac family small GTPase 1 pseudogene 2	51.8	7.9E-05
6	CTNNA3	Catenin alpha 3	25.7	2.2E-09
7	LCN2	Lipocalin 2	25.0	1.1E-09
8	RPSAP44	Ribosomal protein SA pseudogene 44	15.1	5.4E-06
9	MUC2	Mucin 2, oligomeric mucus/gel-forming	14.8	1.2E-02
10	TRPV6	Transient receptor potential cation channel subfamily V member 6	14.0	1.3E-10
11	SPX	Spexin hormone	14.0	2.2E-14
12	PAPPA2	Pappalysin 2	13.5	3.4E-08
13	C8orf44 SGK3/SGK3	Serum/glucocorticoid regulated kinase family member 3	13.4	2.9E-03
14	INHBE	Inhibin subunit beta E	9.1	1.3E-13
15	CPLX3	Complexin 3	9.0	1.2E-02
16	CTAGE3P	CTAGE family member 3, pseudogene	8.8	1.7E-02
17	ROBO4	Roundabout guidance receptor 4	7.4	1.3E-06
18	CLIC4P1	Chloride intracellular channel 4 pseudogene 1	7.4	4.4E-02
19	ZNF322P1	Zinc finger protein 322 pseudogene 1	7.3	9.8E-03
20	ADTRP	Androgen dependent TFPI regulating protein	7.1	6.5E-04
21	PCDHGB3	Protocadherin gamma subfamily B, 3	6.7	1.4E-02
22	PRPH	Peripherin	6.6	2.9E-08
23	PTPDC1	Protein tyrosine phosphatase domain containing 1	6.5	8.8E-15
24	SAA1	Serum amyloid A1	6.5	1.3E-02
25	TBX2	T-box transcription factor 2	6.0	4.1E-04
26	B3GALT5	Beta-1,3-galactosyltransferase 5	5.9	1.2E-09
27	ERICH2	Glutamate rich 2	5.7	5.3E-08
28	LAMP3	Lysosomal associated membrane protein 3	5.5	1.5E-09
29	OR2A20P	Olfactory receptor family 2 subfamily A member 20 pseudogene	5.3	1.6E-06
30	RPL23AP60	Ribosomal protein L23a pseudogene 60	5.1	3.7E-04
31	OR2A9P	Olfactory receptor family 2 subfamily A member 9 pseudogene	5.0	2.5E-12
32	INSC	INSC spindle orientation adaptor protein	4.9	4.0E-05
33	ATP2C2	ATPase secretory pathway Ca2+ transporting 2	4.9	7.8E-08
34	LRP1B	LDL receptor related protein 1B	4.8	5.1E-15
35	PRELID1P4	PRELID1 pseudogene 4	4.6	7.8E-03
36	PCED1B	PC-esterase domain containing 1B	4.6	4.8E-04
37	RGPD4	RANBP2 like and GRIP domain containing 5	4.5	6.3E-08
38	HOXD3	Homeobox D3	4.5	4.6E-02
39	PABPC1L	Poly(A) binding protein cytoplasmic 1 like	4.5	2.2E-08
40	RGMB-AS1	RGMB antisense RNA 1	4.5	3.4E-07
41	CERS6-AS1	CERS6 antisense RNA 1	4.4	2.8E-05
42	ANKRD22	Ankyrin repeat domain 22	4.4	2.5E-03
43	GALNT13	Polypeptide N-acetylgalactosaminyltransferase 13	4.3	1.9E-04
44	ACRBP	Acrosin binding protein	4.2	2.4E-04
45	ASIC4	Acid sensing ion channel subunit family member 4	4.2	1.5E-03
46	SYNE1	Spectrin repeat containing nuclear envelope protein 1	4.0	2.7E-07
47	SPRY4-IT1	SPRY4 intronic transcript 1	4.0	6.7E-08
48	MAGOH2P	Mago homolog 2, pseudogene	3.9	5.9E-03
49	NELL2	Neural EGFL like 2	3.9	1.1E-14
50	ABCC3	ATP binding cassette subfamily C member 3	3.8	4.2E-11

Supplemental S4

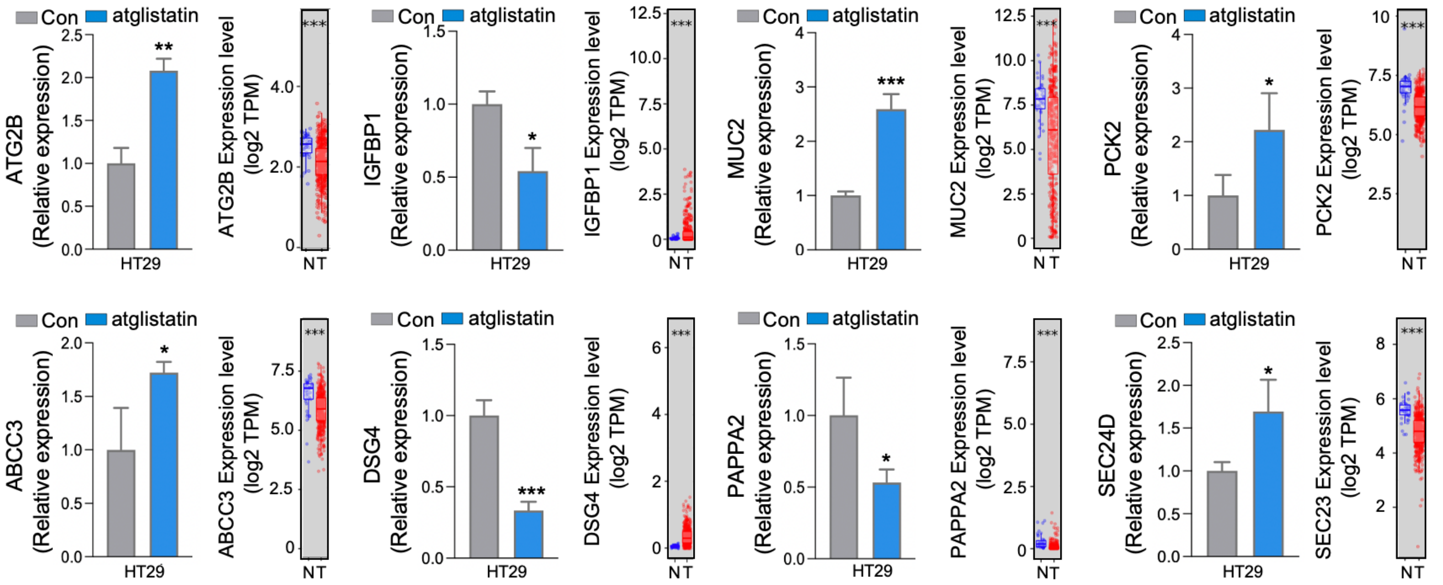

Supplemental S4. Validation of ATGL-mediated differentially expressed genes in colon cancer HT29 cells. HT29 cells were treated with atglistatin (24h) and expression of following transcripts was assessed: ATG2B, IGFPBP1, MUC2, PCK2, ABCC3, DSG4, PAPPA2, SEC24D (part of SEC23 family) (qPCR, n=3, *p<0.05, **p<0.01, ***p<0.001). Similar significant alterations of these transcripts were found in colon cancer patient tissue (grey box plots) (TCGA: normal colon (n=41) and tumors (n=457)).

Supplemental S5

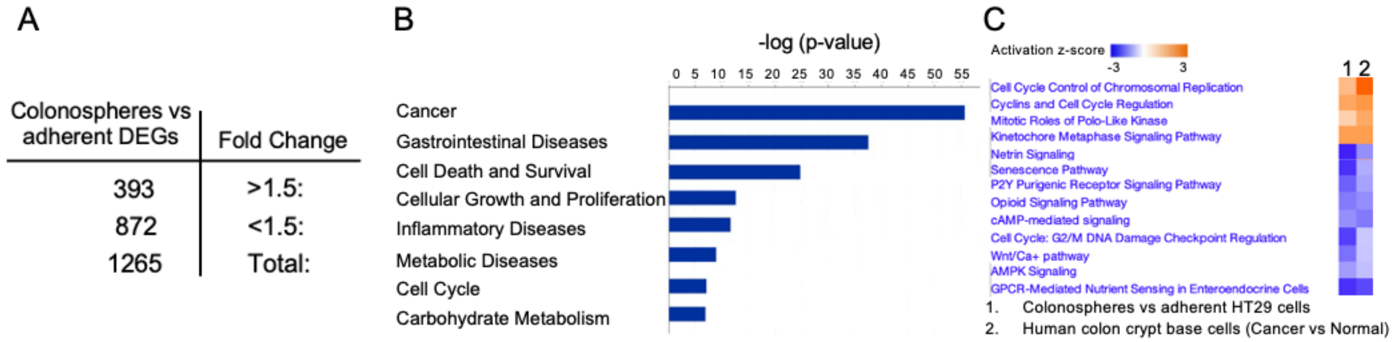

Supplemental S5. Transcriptional differences between colonospheres and adherent colon cancer cells. A) Differentially expressed genes (DEGs) from HT29 colonospheres relative to adherent HT29 monolayers (n=3 for each group, FC >|1.5|, FDR<0.05). B) Top diseases and functions in colonospheres relative to adherent HT29 cells (p<0.05, IPA). C) Similarly altered canonical pathways in colonospheres (vs adherent cells) and human colonic tumor crypt base cells (vs normal crypts) enriched in cancer stem cells from colon cancer patients (GSE20916, p<0.05, IPA).

Supplemental S6

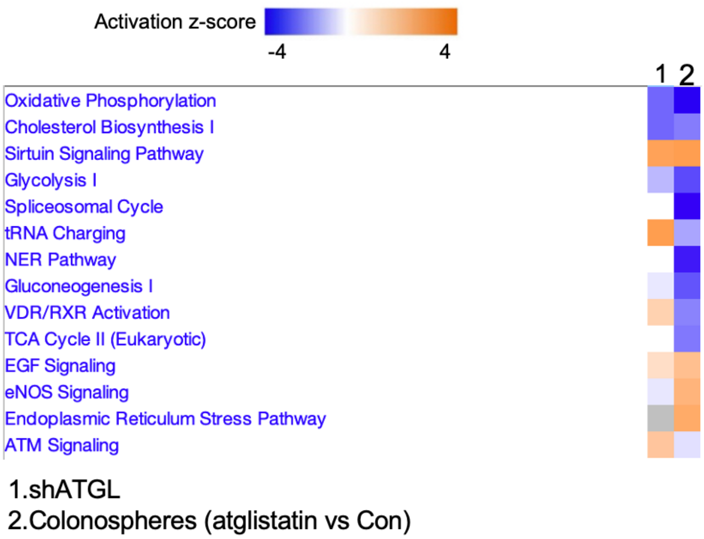

Supplemental S6. Shared and distinct pathways alteration with ATGL inhibition in adherent colon cancer HCT116 cells (clones shATGL) and HT29 colonospheres (atglistatin) ($p<0.05$, IPA).

Supplemental S7: Top fifty differentially expressed genes (DEGs) in human colonospheres (HT29) with ATGL inhibition relative to control (n=3, FC ≥|1.5|, p<0.05).

	Gene	Gene Name	FC	Adj.P.Val
**1**	CYP1A1	Cytochrome P450 Family 1 Subfamily A Member 1	11.1	2.8E-82
**2**	ABCG1	ATP Binding Cassette Subfamily G Member 1	5.2	8.8E-87
**3**	ABCA1	ATP Binding Cassette Subfamily A Member 1	4.5	5.8E-40
**4**	BNIP5	BCL2 Interacting Protein 5	3.5	3.0E-03
**5**	POU2F3	POU Class 2 Homeobox 3	3.4	4.7E-04
**6**	BMF	Bcl2 Modifying Factor	2.7	3.1E-27
**7**	IFNE	Interferon Alpha 1	2.7	3.8E-03
**8**	CYP1B1	Cytochrome P450 Family 1 Subfamily B Member 1	2.6	5.5E-75
**9**	SERPINA3	Serpin Family A Member 3	2.5	3.0E-03
**10**	ALDH1A3	Aldehyde Dehydrogenase 1 Family Member A3	2.5	1.7E-08
**11**	GDF15	Growth Differentiation Factor 15	2.5	7.2E-84
**12**	TP53INP1	Tumor Protein P53 Inducible Nuclear Protein 1	2.4	7.5E-10
**13**	BTBD8	BTB Domain Containing 8	2.3	5.1E-04
**14**	TMEM140	Transmembrane Protein 140	2.3	8.9E-05
**15**	NR0B2	Nuclear Receptor Subfamily 0 Group B Member 2	2.2	1.8E-02
**16**	ATF3	Activating Transcription Factor 3	2.2	1.1E-11
**17**	PIM1	Pim-1 Proto-Oncogene, Serine/Threonine Kinase	2.1	1.3E-11
**18**	PDK4	Pyruvate Dehydrogenase Kinase 4	2.0	9.0E-16
**19**	RN7SL2	RNA Component of Signal Recognition Particle 7SL2	2.0	4.7E-03
**20**	MERTK	MER Proto-Oncogene, Tyrosine Kinase	2.0	3.5E-02
**21**	TCP11L2	T-Complex 11 Like 2	2.0	7.0E-09
**22**	MYLIP	Myosin Regulatory Light Chain Interacting Protein	2.0	8.2E-14
**23**	H4C15	H4 Clustered Histone 15	1.9	3.5E-02
**24**	MMP14	Matrix Metallopeptidase 14	1.9	1.9E-03
**25**	FAM106A	Family With Sequence Similarity 106 Member A	1.9	8.0E-05
**26**	DHRS3	Dehydrogenase/Reductase 3	1.9	6.4E-26
**27**	SLC6A9	Solute Carrier Family 6 Member 9	1.9	8.9E-05
**28**	MYO7A	Myosin VIIA	1.9	1.1E-02
**29**	TMCC3	Transmembrane And Coiled-Coil Domain Family 3	1.9	3.7E-11
**30**	WNT2B	Wnt Family Member 2B	1.9	3.3E-02
**31**	KLHL24	Kelch Like Family Member 24	1.9	1.6E-18
**32**	GABRP	Gamma-Aminobutyric Acid Type A Receptor Subunit Pi	1.9	2.5E-06
**33**	IRS2	Insulin Receptor Substrate 2	1.9	3.6E-11
**34**	RCN3	Reticulocalbin 3	1.9	1.1E-02
**35**	CYP4F3	Cytochrome P450 Family 4 Subfamily F Member 3	1.9	7.8E-03
**36**	STRA6	Signaling Receptor and Transporter Of Retinol STRA6	1.8	1.7E-06
**37**	FAM229A	Family With Sequence Similarity 229 Member A	1.8	4.0E-02
**38**	JUND	JunD Proto-Oncogene, AP-1 Transcription Factor Subunit	1.8	5.4E-33
**39**	SREBF1	Sterol Regulatory Element Binding Transcription Factor 1	1.8	3.7E-24
**40**	BBC3	BCL2 Binding Component 3	1.8	2.4E-16
**41**	NR1H4	Nuclear Receptor Subfamily 1 Group H Member 4	1.8	4.8E-02
**42**	RNF145	Ring Finger Protein 145	1.8	1.2E-35
**43**	SLC38A4	Solute Carrier Family 38 Member 4	1.8	5.5E-13
**44**	ABTB1	Ankyrin Repeat and BTB Domain Containing 1	1.8	6.3E-05
**45**	DEPP1	DEPP1 Autophagy Regulator	1.8	3.9E-03
**46**	MYT1	Myelin Transcription Factor 1	1.8	1.7E-02
**47**	BTBD19	BTB Domain Containing 19	1.8	9.5E-03
**48**	DNHD1	Dynein Heavy Chain Domain 1	1.8	1.6E-03
**49**	CHAC1	ChaC Glutathione Specific Gamma-Glutamylcyclotransferase 1	1.8	1.9E-06
**50**	CYP1B1-AS1	CYP1B1 Antisense RNA 1	1.8	3.5E-02

Supplemental S8

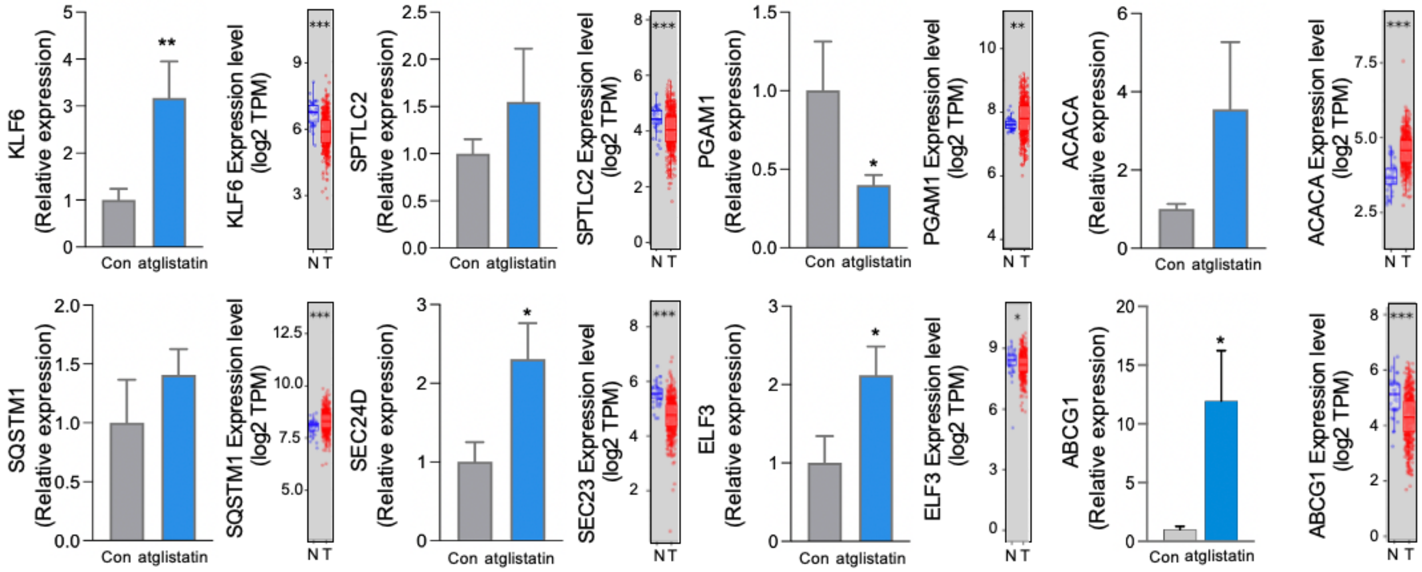

Supplemental S8. Validation of ATGL-mediated differentially expressed genes in colonospheres derived from HCT116 cells. Colonospheres of HCT116 were treated with atglistatin (24h) and expression of following transcripts was assessed: SPTLC2, PGAM1, ACACA, SQSTM1, SEC24D (part of SEC23 family), ELF3 and ABCG1 transcripts in HCT116 colonospheres (qPCR, n=3, *p<0.05, **p<0.01). Similar significant alteration of these transcripts was found in colon cancer patient tissue (grey box plots) with the exception of SQSTM1 (we speculate due to its dual role in autophagy and NF-kB signaling (Sci Rep,2019; Nat Commun,2021) (TCGA: normal colon (n=41) and tumors (n=457)).
